# Clinical Response to Anti-CD47 Immunotherapy Is Associated with Rapid Reduction of Exhausted Bystander CD4^+^ BTLA^+^ T Cells in Tumor Microenvironment of Mycosis Fungoides

**DOI:** 10.3390/cancers13235982

**Published:** 2021-11-28

**Authors:** Tony T. Jiang, Oleg Kruglov, Gloria H. Y. Lin, Angela Minic, Kimberly Jordan, Robert A. Uger, Mark Wong, Yaping Shou, Oleg E. Akilov

**Affiliations:** 1 Cutaneous Lymphoma Program, Department of Dermatology, University of Pittsburgh, Pittsburgh, PA 15261, USA; jiangt2@upmc.edu (T.T.J.); olk19@pitt.edu (O.K.); 2 Trillium Therapeutics Inc., Mississauga, ON L5L 1J9, Canada; gloria@trilliumtherapeutics.com (G.H.Y.L.); bob@trilliumtherapeutics.com (R.A.U.); mark@trilliumtherapeutics.com (M.W.); yaping@trilliumtherapeutics.com (Y.S.); 3 Department of Immunology and Microbiology, University of Colorado, Aurora, CO 80045, USA; angela.minic@cuanschutz.edu (A.M.); kimberly.jordan@cuanschutz.edu (K.J.)

**Keywords:** mycosis fungoides, NK cells, cytotoxic, lymphoma, immunotherapy

## Abstract

**Simple Summary:**

The identification of the events that accompany cancer progression is essential for developing new therapies. We have used mycosis fungoides, the most common type of cutaneous lymphoma, as a model for our study. We have shown that cancer progression is accompanied by the expansion of exhausted immune cells around malignant cells. Those exhausted cells prevent immune activation, blocking cancer clearance by the immune system. Furthermore, we have demonstrated that novel anti-CD47 immunotherapy with mycosis fungoides leads to the reduction of exhausted T cells accompanied by the expansion of NK and CD8^+^ T cells. These therapeutic benefits of CD47 blockade were further facilitated by interferon-α, which stimulates cytotoxic cells. Thus, we showed that CD47 might serve as an effective therapeutic target in treating mycosis fungoides.

**Abstract:**

Cancer progression in mycosis fungoides, the most common form of cutaneous T-cell lymphoma, occurs in a predictable, sequential pattern that starts from patches and that evolves to plaques and later to tumors. Therefore, unlocking the relationship between the microarchitecture of mycosis fungoides and the clinical counterparts of that microstructure represents important steps for the design of targeted therapies. Using multispectral fluorescent imaging, we show that the progression of mycosis fungoides from plaque to tumor parallels the cutaneous expansion of the malignant CD4^+^ T cells that express TOX. The density of exhausted BTLA^+^ CD4^+^ T cells around malignant CD4^+^TOX^+^ cells was higher in tumors than it was in plaques, suggesting that undesired safeguards are in place within the tumor microenvironment that prevent immune activation and subsequent cancer eradication. Overriding the CD47 checkpoint with an intralesional SIRPαFc fusion decoy receptor induced the resolution of mycosis fungoides in patients that paralleled an amplified expansion of NK and CD8^+^ T cells in addition to a reduction of the exhausted BTLA^+^ CD4^+^ T cells that were engaged in promiscuous intercellular interactions. These therapeutic benefits of the CD47 blockade were further unleashed by adjuvant interferon-α, which stimulates cytotoxic cells, underscoring the importance of an inflamed microenvironment in facilitating the response to immunotherapy. Collectively, these findings support CD47 as a therapeutic target in treating mycosis fungoides and demonstrate a synergistic role of interferon-α in exploiting these clinical benefits.

## 1. Introduction

Cutaneous T-cell lymphomas (CTCL) are a heterogeneous group of neoplasms that originate and present in the skin. The most common type of CTCL is mycosis fungoides (MF), which accounts for approximately half of all primary cutaneous lymphomas [1]. Very early stage MF exhibits an indolent course, as appropriately treated patients have lifespans that are comparable to unaffected age-matched controls [2]. However, 15–20% of MF patients inevitably experience disease progression, and those who reach terminal stage IV have a median survival of 18 months despite receiving multi-agent chemotherapies [3]. Thus, there is a pressing need for both effective and well-tolerated therapeutics for MF.

The initial success of immunotherapy in solid cancers has generated widespread interest in their applicability to other malignancies. Although PD-1 blockade has demonstrated efficacy and has been awarded FDA approval for advanced solid tumors, their potency in MF has been less desirable. A phase II trial using the PD-1 inhibitor pembrolizumab demonstrated an ~8% complete response and a 30% partial response in MF patients, with approximately half of the patients with advanced-stage disease experiencing a transient worsening of their symptoms [4]. Similar results were shown in a phase I trial using the anti-PD-1 monoclonal antibody nivolumab, with only 15% of MF patients (2 out of 13) exhibiting a partial response [5]. These findings suggest that the search for immune checkpoint therapy for cutaneous T-cell malignancies must continue.

The failure to respond to immune checkpoint inhibitors stems from pre-existing and/or acquired aberrations in the tumor microenvironment (TME) [6]. These findings were initially detected by identifying genetic mutations or shifts in protein expression among malignant tissue en masse. More recently, the development and application of multiplex immunofluorescent technology have unveiled an additional spatial dimension, which would be otherwise undetected, in understanding how the tumor microarchitecture dictates outcomes to therapy [7]. Using this approach, tumor infiltrating lymphocytes (TILs) within the immediate vicinity of malignant cells in classic Hodgkin lymphoma were shown to be enriched for CTLA-4 using this approach [8], which could explain the proclivity for these patients to develop resistance to PD-1 checkpoint blockade. However, the broader applicability of this approach for cutaneous malignancies remains incompletely explored. 

Prior studies utilizing multispectral imaging for cutaneous T cell lymphomas have focused on the upregulation of checkpoint molecules including PD-1, LAG3, and CTLA-4 among expanded CD4^+^ T cells [9]. While these inhibitory pathways were found to be expressed among exhausted T cells within the lesional skin of CTCL patients, it remains unclear why overriding these molecules does not lead to consistent disease improvement. Therefore, we employed multispectral imaging to visualize and identify the relevance of how other targets of immunotherapy interact within the MF TME. Our goal was to identify changes in the tumor microarchitecture that were associated with clinical responsiveness and to exploit these observations in order to rationally augment modulatory immune therapies in CTCL. 

## 2. Materials and Methods

### 2.1. Patients Treated with TTI-621

Patients with relapsed/refractory MF were treated with TTI-621, a fusion protein composed of the CD47-binding domain, human SIRPα, fused to human IgG1 Fc as part of a phase 1a/1b dose escalation and expansion trial (Clinicaltrials.gov, NCT02890368). Patients MF09 and MF10 did not participate in this clinical trial (their samples were collected as a part of Tissue Banking Protocol, Clinicaltrials.gov, NCT00177268, and were used for a single cell RNA sequencing). Patient characteristics are detailed in Table 1. The study was performed following the principles of the Declaration of Helsinki and the Good Clinical Practice guidelines. 

Eligible patients had percutaneously accessible and histologically confirmed MF tumors; progressed on standard anticancer therapy or had no approved conventional therapy; an Eastern Cooperative Oncology Group performance score of 2 or less; adequate coagulation, hepatic, and renal function; and adequate hematologic status. 

The exclusion criteria included graft-versus-host disease (except for grade 1 skin involvement); prior hemolytic anemia, bleeding diathesis, or coagulopathy; uncontrolled infection; investigational or anticancer therapy within 14 days or major surgery within 21 days before treatment initiation; antiplatelet, anticoagulant, or non-steroidal anti-inflammatory drug within 7 days before treatment initiation until 30 days after treatment; and hematopoietic growth factor within 7 days before treatment initiation.

Patients received six intra-tumoral injections of 10 mg TTI-621 three times per week. Patients received treatment until disease progression, intolerable toxicity, withdrawal of consent, or investigator decision. Clinical response was assessed at weeks 4, 8, 12, and 20 weeks after dosing using the Composite Assessment of Index Lesion Severity (CAILS), with end of treatment (EOT) outcome measured 7 days after the last injection. Patients who had a ≥50% decrease in the CAILS score were deemed “responders”, while patients with a <50% decrease in the CAILS score were deemed “non-responders”. Punch biopsies of the tumor that were 4 mm in size were collected before TTI-621 injection, at maximum induration, and at EOT. The sample collection at the time of the maximal induration was optional per protocol.

### 2.2. Multispectral Fluorescence Immunohistochemistry

Human tumor tissue was fixed in formalin and paraffin-embedded for multispectral imaging on a Vectra Polaris Automated Quantitative Pathology Imaging System (Perkin Elmer, Waltham, MA, USA). Sections that were four microns in size were mounted on glass slides and were sequentially stained for human CD3 (RRID:AB_563541), CD4 (RRID:AB_563559), CD8 (RRID:AB_442067), CD56 (RRID:AB_563905), granzyme B (RRID:AB_442095), TOX (HPAO18322, Sigma-Aldrich, Burlington, MA, USA), BTLA (NBP2-45549, NovusBio, Littleton, CO, USA), and DAPI on a Bond RX autostainer (Leica, Wetzlar, Germany). Slides were dewaxed, heat-treated in Leica Epitope Retrieval Solution 2 antigen retrieval buffer for 20 min at 93 °C, blocked in antibody diluent (Perkin Elmer, Waltham, MA, USA), and incubated for 30 min with primary antibody, for 10 min with horseradish peroxidase-conjugated secondary polymer (anti-mouse/anti-rabbit, Perkin Elmer, Waltham, MA, USA), and for 10 min with horseradish peroxidase-reactive OPAL fluorescent reagent (Perkin Elmer, Waltham, MA, USA). Slides were washed between staining steps with Leica Bond Wash and were stripped between each round of staining with heat treatment in antigen retrieval buffer. After final staining, the slides were heat-treated in Leica Epitope Retrieval Solution 1 antigen retrieval buffer, stained with spectral 4′,6-diamidino-2-phenylindole (Perkin Elmer, Waltham, MA, USA), and coverslipped with Prolong Diamond mounting media (Thermo Fisher, Waltham, MA, USA). Whole slides were scanned using a 10× objective at a resolution of 1.0 μm. Approximately 4–10 regions of interest were chosen in areas either near the dermal–epidermal junction or in the center of the tumor. Regions of interest were scanned for multispectral imaging with a 20× objective at a resolution of 0.5 μm.

### 2.3. Cell Identification

After image acquisition, multispectral images were spectrally unmixed with inForm software (RRID:SCR_019155, Perkin Elmer, Waltham, MA, USA). QuPath software (RRID:SCR_018257) and MATLAB (RRID:SCR_001622, MathWorks, Natick, MA, USA) was used to segment cells based on adaptive thresholding, an approach wherein a different threshold value is computed for each pixel in the image. Bradley’s method, which computes a locally adaptive threshold for each pixel using the local mean intensity around the pixel neighborhood, was chosen due to its robust sensitivity to illumination changes in images [10]. Additional image processing was performed to reduce noise by filtering the image such that objects with a small number of pixels that likely represent artifacts were removed. To separate overlapping cells, a watershed transformation was applied that is able to identify where two cells are connected by analyzing the topography of the pixels. Cellular phenotypes were established based on the combination of positive and negative biomarker staining. Markers were considered to be co-stained if their bounding boxes overlapped by 80%, an amount that we reasoned would allow for nucleic and cytoplasmic staining to identify the same cell. Thresholds for “positive” staining and the accuracy of phenotypic algorithms were confirmed by visual inspection. 

### 2.4. TruSeq Tissue RNA Expression Analysis

Raw TruSeq FASTQ files of CTCL skin lesions were downloaded from SRP114956 [11,12]. Additional clinical data on patient age and disease were downloaded from the SRA repository. FASTQ files were pseudo-aligned with kallisto using the GRCh38 build of the human transcriptome [13]. Transcript-level quantifications were condensed to gene-level and were scaled to transcripts-per-million values.

### 2.5. Single Cell Targeted Transcriptome Sequencing

Single cell suspensions were generated from 1 cm skin punch biopsies of one patient with plaque stage and three patients with tumor stage CD4^+^ MF using a Whole Skin Dissociation Kit (Miltenyi Biotec Inc., Auburn, CA, USA). All samples were adjusted to 1600 cells per sample prior to loading onto a BD Rhapsody Cartridge in the BD Rhapsody Express Single-Cell Analysis System (BD Bioscience, San Jose, CA, USA) according to the manufacturer’s protocol. Single-cell reverse transcription and exonuclease I treatment were performed per BD Rhapsody protocols. To generate libraries, BD Rhapsody mRNA and Sample Tag were encoded on Cell Capture Beads and were amplified in PCR1 along with the BD Rhapsody Immune Response Panel (utilizes multiplex PCR for detecting 399 genes chosen for profiling human immune cells). Sample Tag products were separated from the mRNA-targeted PCR1 products by means of double-sided size selection with Agencourt AMPure XP magnetic beads. Sample Tag PCR1 products underwent a separate index PCR from the mRNA products using library index primers. Thus, each single cell was assigned several unique barcodes to label the cell and the patient to whom the sample belonged (Sample Tag) for a series of pulled NGS targeting 20,000 read depth per cell in a 75-bp × 2 pair-ended fashion on NextSeq 500 (Illumina, San Diego, CA, USA).

The Seven Bridges Genomic Platform (BD Rhapsody Bioinformatics) was used to run a pipeline to quantify the total numbers of molecules of specific genes present per cell using distribution-based error corrections. Based on gene average expressions and dispersions, highly variable genes were identified, and principal component analysis was performed on the scaled data of these variable genes. Cells were clustered by Seurat (Louvain clustering) [14] and were visualized using a uniform manifold approximation and projection (UMAP). The identity of each UMAP cluster was determined by comparing the expression of the most differentially expressed genes. The ligand–receptor interaction analysis between malignant and tumor-infiltrating cells was conducted through the CellPhoneDB database [15].

### 2.6. Gene Set Enrichment Analysis (GSEA)

GSEA was performed using GSEA software v4.1.0 [16]. We estimated the signal-to-noise ratio and false discovery rates (FDR) from 1000 gene-set permutations due to the low number of samples in each class.

### 2.7. Statistical Analysis

Statistical tests were performed using GraphPad Prism 9 (RRID:SCR_002798). Differences between the two groups were compared by the two-tailed unpaired Student’s *t*-test without the assumption of equal variance (Welch’s correction). When more than two groups were compared with one another, the one-way ANOVA with Sidak–Holm’s post-test correction was employed. Statistical significance was determined as *p* < 0.05.

## 3. Results

### 3.1. TOX^+^CD4^+^ T Cell Density Correlates with MF Progression

**Figure 1:**
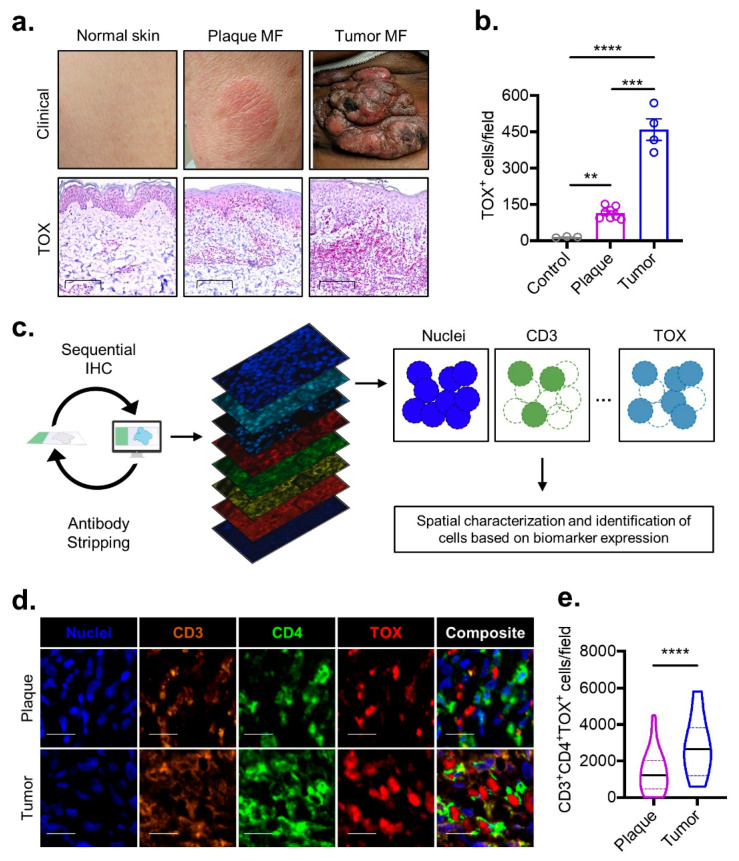
TOX density correlates with MF disease stage. (**a**) Representative clinical images (top) and high-powered immunohistochemical TOX staining (bottom, scale bar 100 µm) for non-neoplastic normal skin compared to plaque and tumor stage MF. (**b**) Composite data illustrating the number of TOX-positive cells per field for the groups described in subfigure (**a**) (*n* = 3 control, *n* = 7 plaques, *n* = 4 tumors). (**c**) Experimental schematic demonstrating the pipeline for employing multispectral imaging to quantify cells of a specific phenotype. Samples undergo sequential immunohistochemical staining for a panel of several biomarkers followed by computer vision-based analysis to locate and identify cell types. (**d**) Multispectral imaging (scale bar 20 µm) from plaque and tumor stage MF stained with the indicated biomarkers. (**e**) Violin plots of composite data enumerating density of CD3^+^CD4^+^TOX^+^ cells per multispectral field for pooled plaque (*n* = 5) or tumor (*n* = 2) MF samples. Bar plots represent mean ± SEM. Violin plots represent the median with the 25th and 75th percentiles. **, *p* < 0.01; ***, *p* < 0.001; ****, *p* < 0.0001 by one-way ANOVA with Sidak–Holm’s correction (panel **b**) or unpaired *t*-test with Welch’s correction (panel **e**).

The expression of the thymocyte selection-associated high mobility group box protein (TOX) has been reported to be enriched among T cells in MF when compared to benign inflammatory conditions [17,18,19,20]. In the differential diagnosis of hypopigmented MF from a clinical mimicker, vitiligo, TOX positivity was reported to be precise with 93.3% sensitivity and 93.9% specificity [21]. While TOX expression has been previously shown to be increased with the progression of MF from patches to plaques and then to tumors [17,22], preceding investigations failed to assess the TOX expression in conjunction with the expression of CD3 and CD4. To address the limitations, we assessed the utility of this oncogenic marker in MF by quantifying TOX protein expression in skin samples from patients with plaque and tumor-stage MF stained simultaneously for CD3, CD4, and CD8 in multispectral sequential fluorescence immunohistochemistry (MSI) by comparing that data with those from healthy controls. A computer-vision-based algorithm was employed to circumvent the biases that stem from manual quantification. The density of the TOX-expressing cells was nearly 10-fold enriched among plaque MF compared to the density of TOX-expression cells in the healthy skin biopsies (Figure 1a,b). The frequency of TOX-expressing cells progressed in parallel with the advancing MF stage, as ~3-fold more TOX-positive cells were identified from tumors compared to from plaques (Figure 1a,b). In order to characterize these malignant cells and their spatial relationship with TILs while overcoming the limitations of classical immunohistochemical staining that preclude simultaneous staining with a large array of biomarkers, we employed multispectral sequential immunofluorescent imaging to track the expression of nine biomarkers at a single-cell resolution (Figure 1c). Our computer vision-based algorithm was refocused to accurately identify the spatial coordinates and staining characteristics of each cell across all of the different biomarkers. Using this approach, we found that the median density of malignant CD4^+^ T-cells that express TOX more than doubled among tumor compared to plaque samples (Figure 1d,e). Collectively, these results reinforced TOX as a biomarker for aberrant CD4^+^ T cells in MF and poised us to investigate the TME surrounding these identifiable malignant cells.

### 3.2. Immunologic Microarchitecture Shifts in MF

**Figure 2:**
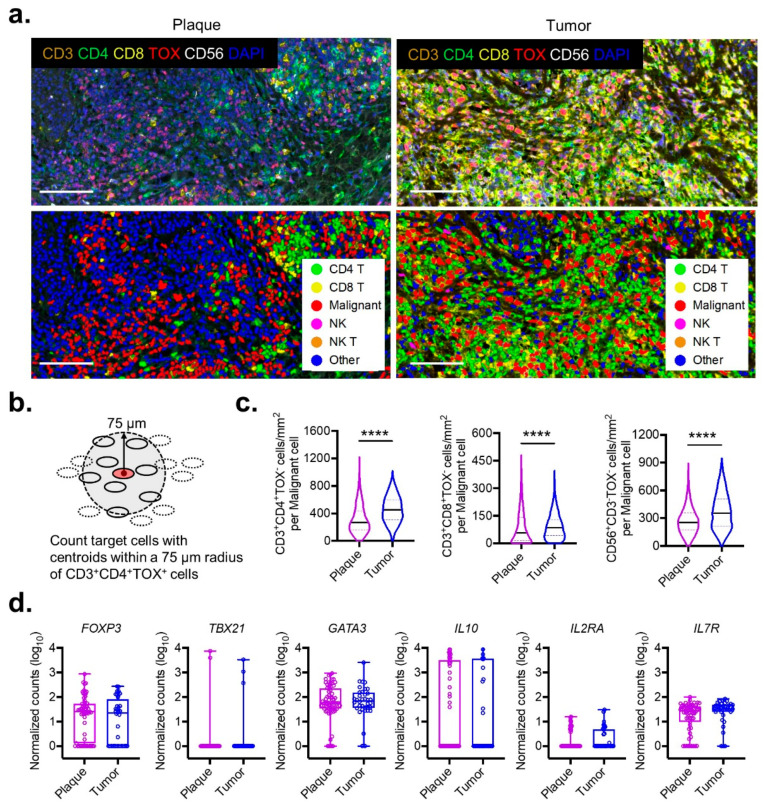
Microarchitecture progression from plaque to tumor stage in MF. (**a**) Representative multispectral images (top) and cell segmentation with phenotype map (bottom) from plaque and tumor stage MF stained with the indicated biomarkers (scale bar 100 µm). (**b**) Schematic diagram for enumerating all cells of a specific phenotype within a 75 μm vicinity of malignant CD3^+^CD4^+^TOX^+^ cells. (**c**) Violin plots of composite data enumerating density of CD3^+^CD4^+^TOX^−^, CD3^+^CD8^+^TOX^−^, and CD56^+^CD3^−^TOX^−^ cells within the vicinity of malignant cells as described in panel (**b**) for pooled plaque (*n* = 5) or tumor (*n* = 2) MF samples. At least five regions of interest were quantified for the analysis. ****, *p* < 0.0001 by unpaired *t*-test with Welch’s correction. (**d**) Normalized tissue RNA expression for the indicated genes between plaque- (*n* = 63) and tumor- (*n* = 36) stage MF samples. Bar plots represent mean ± SEM. Violin plots represent the median with 25th and 75th percentiles.

A striking feature of classic MF is that disease progression will occur in a sequential pattern: from a thin patch to an infiltrative plaque, and then to an exophytic tumor. We reasoned that the most dramatic shifts in TME likely occur in the transition from plaque to tumor, as this change engenders the most precipitous decline in survival [23]. Therefore, the spatial changes imparted by plaque-to-tumor transformations on the microarchitecture of malignant cells was visualized. The analysis of multispectral immunofluorescent imaging showed that the overall cellular density was greater in tumors than it was in plaques (Figure 2a).

To precisely quantify the cellular density around malignant cells, a “vicinity” region was defined as the area within 75 µm of a malignant TOX^+^CD4^+^ T cell—a distance that was shown to be crucial for cell-to-cell interactions in other solid cancers [24] (Figure 2b). The processed outputs from this algorithm demonstrated a sharp influx of TILs in the vicinity of malignant TOX^+^CD4^+^ T cells that was associated with the progression from the plaque to tumor stage. The density of tumor-infiltrating non-malignant TOX^−^CD4^+^ T cells surrounding their malignant TOX^+^ counterparts increased by 68% in tumors compared to plaques (median 453 cells/mm^2^ vs. 269 cells/mm^2^, *p* < 0.0001) (Figure 2c). A similar but slightly less robust expansion was observed for cells with cytotoxic capabilities including CD8^+^ T cells (median 85 cells/mm^2^ vs. 57 cells/mm^2^, *p* < 0.0001) and NK cells (median 354 cells/mm^2^ vs. 255 cells/mm^2^, *p* < 0.0001) (Figure 2b).

The observation that malignant cells in advanced-stage MF could co-exist with a dense lymphocytic infiltrate implicated the presence of additional immune-suppressive safeguards mediating this acquired tolerance. A previous study utilizing the bulk RNA sequencing of patients’ samples with plaque and tumor MF lacked granularity in the characterization of the TME. We analyzed the raw sequencing reads of bulk RNA expression [11,12] from the clinical samples of a cohort of patients with plaque- (*n* = 66) and tumor- (*n* = 36) stage MF (Figure 2d). While the expression levels of *FOXP3* were implicated in the clonal evolution of malignant cells [25], along with other lineage-defining transcription factors and cytokines (*TBX21* for Th1, *GATA3* and *IL10* for Th2), we could not detect any differences between MF plaques and MF tumors. Moreover, no significant differences were found for cellular activation markers, including *IL2RA* and *IL7R*, suggesting that immunologic shifts in the TME escapes detection at the bulk RNA sequencing resolution.

### 3.3. An Expanstion of a Population of Exchausted BTLA^+^ CD4^+^ T Cells from Plaque to Tumor MF

**Figure 3:**
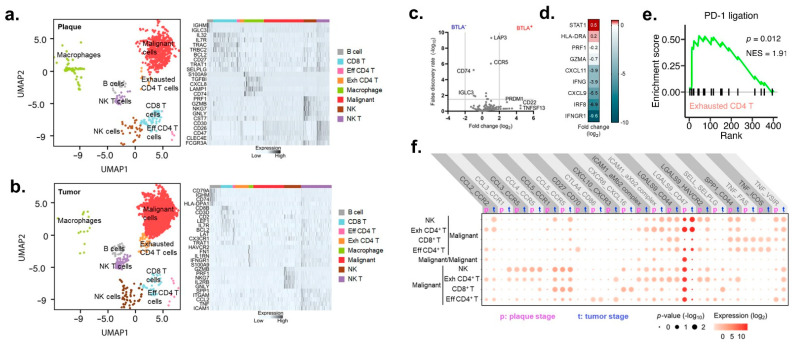
Intercellular interactions between malignant cells and TILs. (**a**) UMAP projection of single-cell RNA sequencing from a patient with plaque-stage mycosis (left). Heat map showing relative expression (*z*-score) of selected genes for cells identified in subfigure (**a**) (right). (**b**) UMAP projection of single-cell RNA sequencing from three patients with tumor-stage mycosis (left). Heat map showing relative expression (*z*-score) of selected genes for cells identified in subfigure (**b**) (right). (**c**) Volcano plot illustrating differential tissue gene expression from BTLA^−^ or BTLA^+^ non-malignant CD4^+^ T cells. (**d**) Changes in IFN-γ genes in exhausted CD4^+^ T cells in comparison with non-exhausted CD4 T-cells. (**e**) Enrichment plots of gene set enrichment analysis for the gene expression profile of exhausted CD4^+^ T cells using PD-1 ligation gene set. (**f**) Intercellular CellPhoneDB analysis illustrating shifts in expression of the indicated ligand-receptor complexes for malignant cells between plaque and tumor stage MF.

To enhance our ability to detect gene expression changes in TME, single-cell RNA sequencing on plaque and tumor MF samples was performed. We observed eight distinct clusters based on an unsupervised UMAP dimensionality reduction in both MF plaques and tumors (Figure 3a,b). The identity of each UMAP cluster was determined by comparing the expression of the most differentially expressed genes. We observed that CD4^+^ T cells readily segregated into exhausted and effector populations. Effector CD4^+^ T cells exhibited enriched expression of the migratory and survival markers BCL2 and CX3CR1. Exhausted CD4^+^ T cells had decreased CD3 expression and increased PRDM1 (PD-1) that reflected chronic activation (Figure 3c) [26]. Importantly, the CD4^+^ T cells that expressed the co-inhibitory molecule BLTA massively downregulated pro-inflammatory cytokines and cytotoxic molecules, including *PRF1*, *GZMA*, *IFNG*, when compared to cells that lacked BLTA expression (Figure 3d). Gene set enrichment analysis of exhausted CD4^+^ T cells from plaque and tumor MF demonstrated the significant upregulation of the PD-1 ligation pathway (Figure 3e), which is in agreement with prior analyses investigating the MF TME [9], further supporting BTLA to be a surrogate marker of exhaustion [27].

Next, the ligand–receptor interaction analysis between malignant and tumor-infiltrating cells was conducted through the CellPhoneDB database [15]. We found 20 pairs of ligands, and their receptors were significantly enriched in both plaque and tumor MF (Figure 3f, Figures S1 and S2). The most prominent complex was found to be the S-type lectin, galectin-9 (*LGALS9*), which has the “do-not-eat-me” surface glycoprotein CD47. Interestingly, with MF progression, the LGALS9-CD47 interaction switches places: in plaque MF, exhausted BTLA^+^ CD4^+^ T cells express CD47, and the tumor cells express ligand LGALS9, but in tumor MF, the opposite occurs. Immunologic participation in complexes with trafficking chemokine receptors CCR2 and CXCR3 became significant in tumor but not plaque MF (Figure 3f), which coincided with the observed influx of TILs observed with multispectral imaging (Figure 2a–c).

### 3.4. Exhausted BTLA^+^ CD4^+^ T Cells in Plaque and Tumor MF

**Figure 4:**
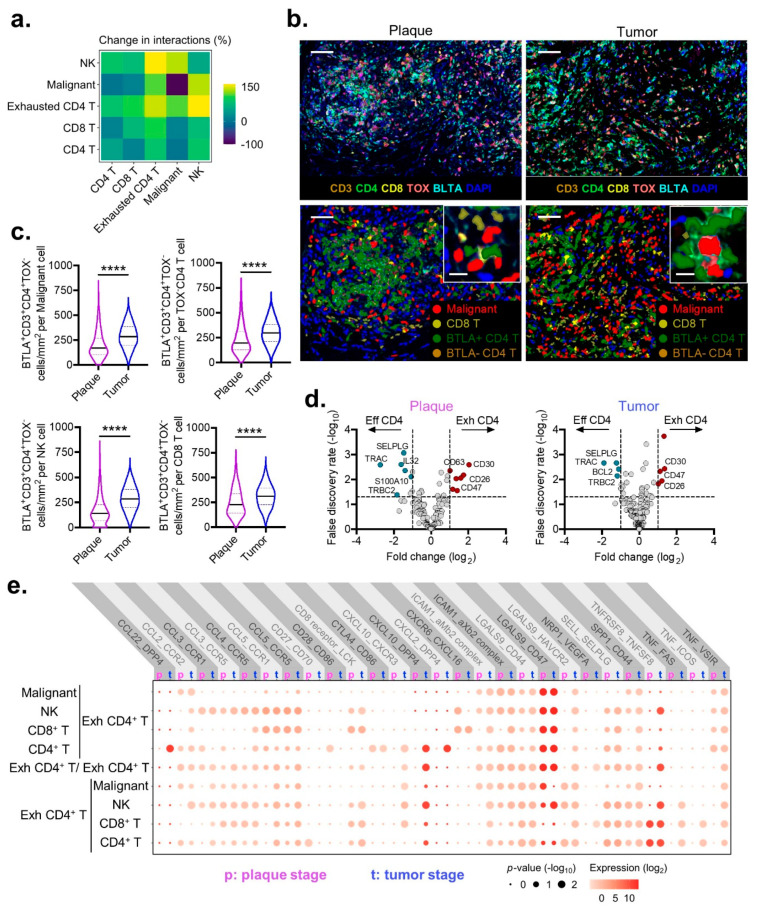
Intercellular interactions between exhausted CD4^+^ T cells and TME. (**a**) Percent change in the number of intercellular interactions between the indicated immune cell types for tumor compared with plaque stage MF. A positive number indicates an increase in interactions from plaque to tumor. (*n* = 1 patient with plaque MF and 3 patients with tumor MF) (**b**) Representative multispectral images from plaque and tumor-stage MF stained with the indicated biomarkers (top) (scale bar 50 µm; insert, scale bar 10 µm). Cell segmentation with phenotype map for malignant, CD8^+^ T, BTLA^+^ CD4^+^ T, and BLTA^−^ CD4^+^ T cells, with inset highlighting spatial arrangement of BTLA^+^ CD4^+^ non-malignant cells in the immediate vicinity of malignant cells (bottom). (**c**) Violin plots of composite data enumerating the density of exhausted BTLA^+^CD3^+^CD4^+^TOX^−^ cells within the immediate 75 um vicinity of malignant and non-malignant CD4^+^ T, NK, and CD8^+^ T cells from pooled plaque- (*n* = 5) or tumor- (*n* = 2) stage MF samples. At least five regions of interest were quantified for the analysis. Violin plots represent the median with 25th and 75th percentiles. ****, *p* < 0.0001 by unpaired *t*-test with Welch’s correction. (**d**) Volcano plots demonstrating differential gene expression between effector and exhausted CD4^+^ T cells for plaque and tumor stage MF samples. (**e**) Intercellular CellPhoneDB analysis illustrating shifts in expression of the indicated ligand–receptor complexes for exhausted cells between plaque and tumor stage MF.

The observation that exhausted CD4^+^ T cells could participate in potential interactions with other TILs and malignant cells, suggesting that they may not be functionally inert but instead biologically relevant in the context of MF. In the transition from plaque MF to tumor MF, exhausted CD4^+^ T cells exhibited the most dramatic increase in ligand–receptor interactions of 122% (Figure 4a). By contrast, malignant cells were the least engaged, with an average overall binding increase of 33% (Figure 4a). To investigate whether exhausted CD4^+^ T cells were in the physical proximity of their theoretical binding partners in TME, multispectral imaging was performed (Figure 4b). The visualization of exhausted BTLA^+^ CD4^+^ T cells in plaques consistently revealed their presence within the vicinity of malignant, NK, CD8^+^, and effector CD4^+^ T cells, and their densities each significantly increased in the transition from plaque MF to tumor MF (Figure 4c).

A comparison of these exhausted CD4^+^ T cells with their effector counterparts demonstrated the significant downregulation of various activation markers, including *BCL2*, *SELPLG*, *S100A10*, and *IL32* (Figure 4d) [28], with the reciprocal upregulation of *CD26* (*DPP4*), CD30 (*TNFRSF8*), and *CD47* that each persisted from plaques to tumors (Figure 4d). Ligand–receptor interactions for exhausted CD4^+^ T cells compared through CellPhoneDB revealed significant intercellular crosstalk with malignant, effector CD4^+^ T, NK, and CD8^+^ T cells (Figure 4e). Uniquely, exhausted CD4^+^ T cells interact with malignant cells via CCR2-CCL2 and the already-mentioned CD47-LGALS9 effector CD4^+^ T via DPP4-CCL22; with NK cells via CXCL10-DPP4 and TNFSF8-TNFRSF8; and with CD8^+^ T cells via CXCL16-CXCR6. These findings are taken in the context that CD26, CD30, and CD47 have been implicated in promoting tumor progression collectively, which suggests that exhausted CD4^+^ T cells participate in the cellular crosstalk that contributes to the pathogenesis of MF [29,30,31,32].

### 3.5. Tumor Microarchitecture following CD47 Blockade

**Figure 5:**
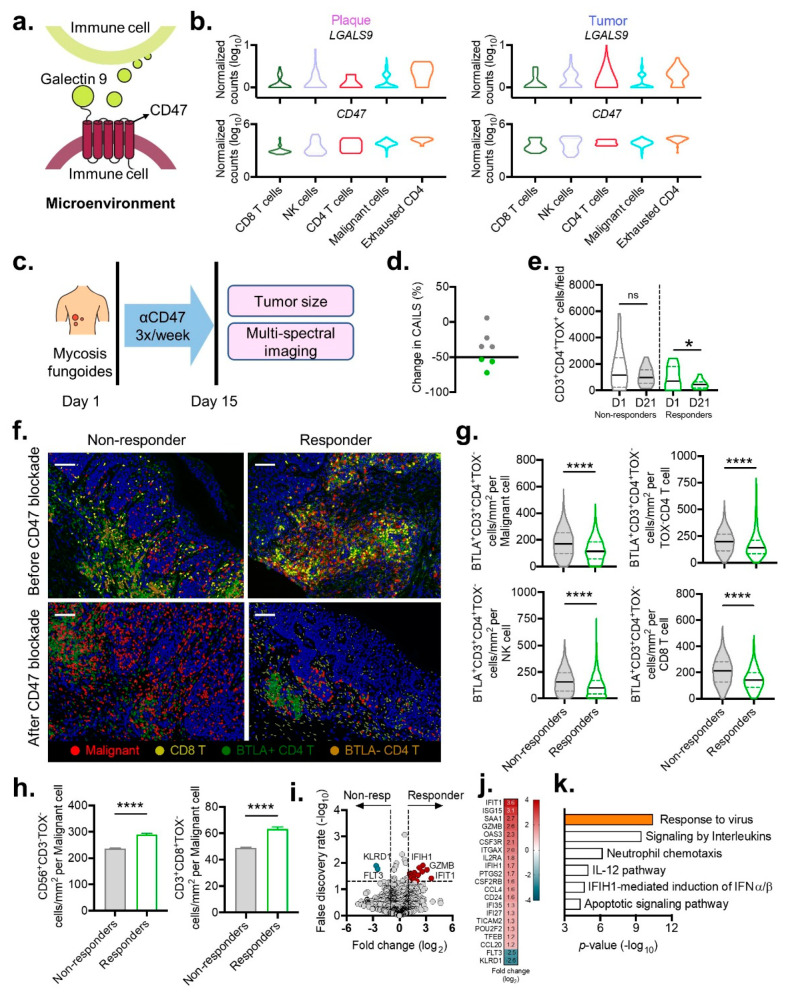
Immunologic shifts within the tumor microenvironment induced by CD47 blockade. (**a**) Model of the Galectin-9-CD47 complex between two immune cells. (**b**) Violin plots show log-transformed, normalized expression levels of the components of the Galectin-9-CD47 complex from plaque (left) and tumor stage (right) MF samples. (**c**) Schematic for intralesional CD47 blockade with a SIRPαFc fusion decoy receptor (TTI-621) three times per week for two weeks in a cohort of 7 patients with MF. (**d**) Percent change in CAILS after treatment with CD47 blockade. Responders with at least 50% decrease in CAILS are labeled in green. Non-responders with less than 50% decrease in CAILS are labeled in gray. (**e**) Violin plots of composite data enumerating density of malignant CD3^+^CD4^+^TOX^+^ cells per field before and after CD47 blockade for responders compared with non-responders. (**f**) Representative cell segmentation with phenotype map for malignant, CD8^+^ T, BTLA^+^ CD4^+^ T, and BLTA^−^ CD4^+^ T cells for responders and non-responders before and after CD47 blockade (scale bar 100 µm). (**g**) Violin plots of composite data enumerating the density of exhausted BTLA^+^CD3^+^CD4^+^TOX^−^ cells within the immediate 75 μm vicinity of malignant and non-malignant CD4^+^ T, NK, and CD8^+^ T cells from responder or non-responders before (top) and after (bottom) CD47 blockade. Violin plots represent the median with 25th and 75th percentiles. *, *p* < 0.05; ****, *p* < 0.0001 by unpaired *t*-test with Welch’s correction. (**h**) Composite data comparing the density of cytotoxic NK (CD56^+^CD3^−^TOX^−^) and CD8^+^ T (CD3^+^CD8^+^TOX^−^) cell density within the immediate 75 μm vicinity of malignant cells from responder or non-responder clinical samples. (**i**) Volcano plot illustrating differential tissue gene expression from responder or non-responder clinical samples. (**j**) Statistically significant genes up- and downregulated in responders. (**k**) Metascape gene enrichment analysis for significantly upregulated genes identified in subfigure (**j**).

Malignant lymphocytes widely express CD47 to promote their survival and evade immune surveillance [32,33]. Interestingly, we found exhausted CD4^+^ T cells expressed not only high levels of CD47 but also the ligand Galectin-9 in the TME of both plaque and tumor MF (Figure 5a,b). Therefore, we reasoned that the CD47 blockade that depletes CD47^+^ cells would simultaneously eliminate any potential Galectin–9:CD47 interactions. To this end, we explored the topography of the TME before and after intralesional CD47 blockade with the SIRPαFc fusion decoy receptor in a cohort of patients experiencing disease progression on conventional therapies (Figure 5c). Response to treatment was measured by the composite assessment of index lesion severity (CAILS), which incorporates erythema, scaling, thickness, pigmentation, and size [34]. In our cohort, three out of seven patients exhibited clinical regression, which was defined as a ≥50% decrease in CAILS, with the remaining patients who did not respond to therapy being labeled as non-responders (Figure 5d).

Clinical outcome paralleled shifts in the microarchitecture. Compared to baseline, the responders demonstrated an ~2-fold reduction in the density of the TOX^+^CD4^+^ T cells (from 958 ± 890 (mean ± SD) cells/field to 453 ± 346 cells/field, *p* = 0.04) (Figure 5e). This contrasted with the non-responders, who displayed no significant change in malignant cell density (mean 1604 ± 1563 cells/field vs. 1076 ± 676 cells/field, *p* = 0.14) (Figure 5e). Response to treatment was also correlated with a reduction in exhausted CD4^+^ T cells, with significant decreases being observed within the vicinity of malignant TOX^+^CD4^+^ T cells and benign TOX^−^CD4^+^ T cells, NK, and CD8^+^ T cells (Figure 5f,g). Importantly, the depletion of exhausted CD4^+^ T cells coincided with a reciprocal increase in cytotoxic NK and CD8^+^ T cells in the immediate proximity of malignant cells (Figure 5h). Significant differences in RNA expression related to immune cell migration and responsiveness to cytokines between the responders’ skin biopsies and those from the non-responders were also observed (Figure 5i–k). These findings demonstrate the accumulation and activation of cytotoxic cells, with the selective depletion of exhausted cells being associated with responsiveness to CD47 blocking immunotherapy, providing a cellular explanation for why a subset of patients do not exhibit improvement to this treatment.

### 3.6. Synergistic Co-Administration of IFNα with CD47 Blockade

**Figure 6:**
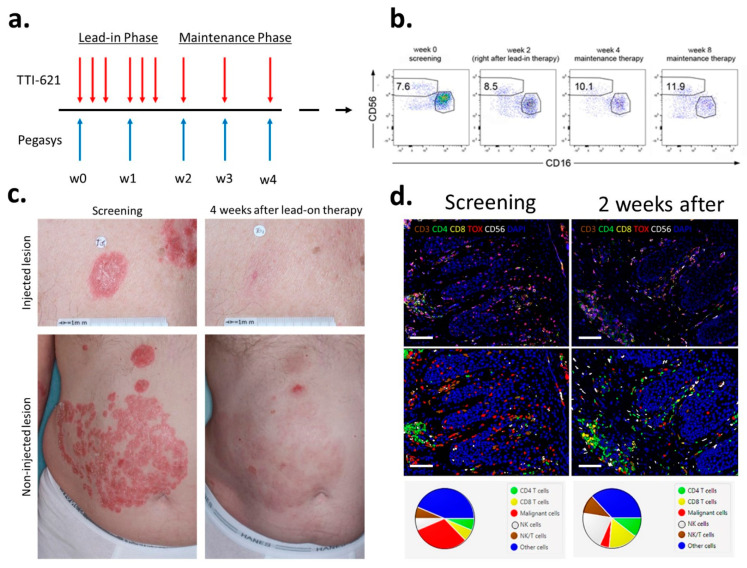
Interferon-α supplementation primes clinical response to CD47 blockade. (**a**) Schematic of the clinical trial design. (**b**) An increase of NK cells (CD3-CD16-CD56^bright^) in the peripheral blood of a representative patient treated with a combination of TTI-621 and pegylated IFN-α2a. Cells gated on CD3^−^. (**c**) Clinical images of a patient with relapsed/refractory tumor MF treated with six intra-tumoral injections of TTI-621 combined with two subcutaneous injections of PEG-IFN-α2a. The patient discontinued the treatment after the lead-in phase. The response is 4 weeks after stopping all the medications. (**d**) Multispectral fluorescent immunohistochemistry of skin biopsies before and 2 weeks after the Lead-In Phase (top panels, scale bar 100 µm). Cell segmentation with phenotype map (bottom panels, scale bar 100 µm) shows as influx of NK cells in TME after treatment and a decrease in the percentage of tumor cells.

Considering that IFNα is FDA approved for the treatment of MF, along with its potent ability to activate cytotoxic cells in the context of antitumor immunity [35], led us to investigate whether IFNα could augment the benefits of CD47 blockade. As a proof of concept, four patients with MF were treated with a combination of TTI-621 and pegylated IFN-α2a via an intra-tumoral injection of 10 mg TTI-621 three times per week for two weeks and 90 mcg pegylated IFN-α2a given subcutaneously weekly (Figure 6a). We compared the CAILs 2 months after the treatment in 11 patients, with 7 patients in the monotherapy cohort (anti-CD47 only) vs. 4 patients in the combination cohort. The overall response rate was 27.2% in the monotherapy cohort (which is in accordance with our published data on a larger cohort of patients [36]) vs. 75% in the combination cohort. Rapid skin clearance was observed within the first 3–4 weeks of a combination therapy with TTI-621 and pegylated IFN-α2a (Figure 6c) in all four patients and was accompanied by an increase of NK cells (CD3-CD16-CD56^bright^) in the peripheral blood (Figure 6b) and in TME (Figure 6d). Collectively, these findings support a role for type I interferons in boosting immune cell responsiveness and clinical outcomes after CD47 blockade.

## 4. Discussion

The results presented here unravel how MF progression results from an accumulation of malignant and exhausted CD4^+^ T cells that exploit the “do-not-eat-me” surface protein, CD47, to evade immune surveillance. CD47 is a ubiquitously expressed membrane receptor of the immunoglobulin superfamily [37] that has been implicated in many normal and pathophysiological processes, including apoptosis, proliferation, cell adhesion, cardiovascular effects, inflammation, and immunity [38]. Disruption of the CD47-SIRPα signaling axis results in the enhanced phagocytosis of both solid [39] and hematopoietic tumor cells [40], leading to significant anti-tumor activity in vivo [41]. The results of phase I clinical trials clearly demonstrated that anti-CD47 blockade enhances antitumor immunity and leads to clinical response in CTCL patients [33,36]. Overriding CD47 while supplementing with FDA-approved IFNα that stimulates cytotoxic cells unleashes tumor clearance.

We established a new mechanism of CD47 blockade that is associated with depletion of exhausted BTLA^+^ CD4^+^ T cells and the reciprocal activation and expansion of cytotoxic CD8^+^ T and NK cells along with the elimination of malignant cells.

BTLA is a type I transmembrane receptor belonging to the Ig-superfamily. In mice, BTLA deficiency is associated with hyper-reactive B and T cells and enhanced susceptibility to autoimmunity [42], which indicates its role as a checkpoint receptor. BTLA is broadly expressed on T cells, and it is not surprising that Sézary cells upregulate BTLA expression on their surface as well [43]. Interestingly, non-clonal bystanders in Sézary syndrome have also been shown to have high BTLA expression that is sometimes even higher than Sézary cells [43]. In concordance with these data, we identified a population of bystander BTLA^+^ CD4^+^ T cells in proximity to tumor cells. Those cells were exhausted, as demonstrated by the downregulation of pro-inflammatory and IFN-γ related cytokines. Upon anti-CD47 treatment, the number of those BTLA^+^ CD4^+^ T cell bystanders decreased dramatically. Based on our data, we propose that BTLA can be a target of tumor immunotherapy alone or in combination with anti-CD47. Recent murine experiments [44] demonstrated that mice treated with anti-BLTA Ab alone showed significant anti-tumor activities that were comparable to those of the cytostatic-treated group.

Among many interactions, besides those of the integrins and signal regulatory protein α (SIRPα), CD47 binds thrombospondin-1, a potent gatekeeper of tumor progression. The TSP1-CD47 signaling pathway promotes tumor progression in MF, and serum TSP1 levels are significantly elevated in patients with Sézary syndrome [45]. Since thrombospondin-1 stimulates cell migration and may potentially contribute to the spread of cancer, blocking TSP1–CD47 interaction may be another novel therapeutic approach in CTCL.

While CD30 expression on malignant cells in MF can be associated with large cell transformation and poor prognosis, the role of by-standing CD30^+^ cells in MF has remained more enigmatic. Our findings reveal that exhausted BTLA^+^ CD4^+^ T cells expressing CD30 can interact with other cells in the TME and that their depletion is associated with cytotoxic cell activation. When taken in the context of early studies reporting CD30 expression to be enriched in disease states and among Th2 polarized human CD4^+^ T cells [46,47,48] and that Th2 cytokines fuel MF progression [49], a logical hypothesis would be that CD30 marks a subset of CD4^+^ T cells that can promote tumorigenesis by indirectly dampening the activation of cytotoxic cells through Th2 microenvironment skewing. This would provide a mechanistic understanding of why CD30 expression is a negative prognostic marker in MF [31,50] and how the targeted depletion of CD30 expressing cells with brentuximab vedotin can induce clinical responses in patients whose malignant cells are CD30 negative [51,52].

The composition of the TME is increasingly appreciated to dictate the response to immunotherapies. We have shown that clinical response to anti-CD47 therapy is accompanied by a surge of cytotoxic NK and CD8^+^ T cells within the immediate vicinity of malignant cells. These inflamed TMEs were also enriched for molecular gene signatures that reflected immune cell migration and activation. Similar relationships have been shown for anti-PD-1/PD-L1 therapy, wherein a “hot” tumor environment is better poised to respond to immune-modulatory therapies [53]. This theory would explain the augmented responses that are observed after CD47 blockade is supplemented with IFNα, which mobilizes cytotoxic cells. Since disease in MF progresses in a distinct sequential pattern, how and where the dichotomy between a hot or cold phenotype develops remains unclear and represents a fertile area for future research.

## 5. Conclusions

Collectively, this work reinforces how the visualization of the TME can be exploited to identify unique immunologic features that may enhance responsiveness to immune-modulatory therapies. This is especially critical in the setting of relapsed/refractory MF, where disease reversal is rarely achieved with conventional chemotherapies. Initial observations demonstrating disease could be halted with extracorporeal photopheresis supplemented with IFNα underscored a potential role of the host immune system in mediating clinical remission. As more immunotherapies develop, we anticipate the visualization of the TME in response to treatment will be instrumental in developing guided approaches to identify potential responders before immunotherapy and in eludicading the mechanisms of drug resistance. Thus, this study serves as the basis for the next generation of targeted treatments that are designed to fine-tune the current immune modulatory modalities.

## Figures and Tables

**Table 1:** Patient characteristics.

Patient	Sex	Diagnosis (Dx)	Age at Dx (y)	Stage at Dx	Age at Sample Collection	TTI-621 Clinical Trial	TNMB at Enrollment	Studies Performed	Prior Treatment(s)
MF01	F	MF	62	IB	70	Yes	T3N0M0B0	MSI, scRNA-seq *	bexarotene, NB-UVB, IFN, LEBT, ECP, brentuximab vedotin
MF02	M	MF	66	IIB	68	Yes	T3N0M0B0	MSI	LEBT
MF03	F	MF	42	IA	64	Yes	T2bN0M0B0	MSI	ECP, chlormethine, PUVA, bexarotene, MTX, pralatrexate, romidepsin
MF04	F	MF	39	IVA1	63	Yes	T3N1M0B0	MSI	ECP, IFN, allogeneic HSCT, LEBT, brentuximab vedotin, vbortezomib
MF05	M	MF	60	IB	63	Yes	T3N1M0B0	MSI	bexarotene, IFN
MF06	M	MF	59	IA	72	Yes	T3N0M0B0	MSI	LEBT, NB-UVB, bexarotene, IFN, chlormethine, pralatrexate
MF07	M	MF	52	IIB	61	Yes	T1bN0M0B0	MSI, scRNA-seq *	LEBT, MTX, bexarotene, chlormethine
MF08	M	MF	72	IB	72	Yes	T1bN0M0B0	MSI	None
MF09	F	MF	66	IIB	72	No	-	scRNA-seq	chlormethine, IFN, bexarotene, brentuximab vedotin, romidepsin, pralatrexate
MF10	F	MF	83	IIB	83	No	-	scRNA-seq	NB-UVB, pralatrexate

* scRNA-seq samples were collected prior to enrollment in the TTI-621 clinical trial. IFN = pegylated interferon alfa-2a. LEBT = low-dose electron beam radiation. ECP = extracorporeal photophoresis. MTX = methotrexate. HSCT = hematopoietic stem cell transplant.

## Data Availability

Single cell RNA-sequencing data will be deposited in Gene Expression Omnibus upon the acceptance of the manuscript.

## Supplementary Materials

The following are available online at https://www.mdpi.com/article/10.3390/cancers13235982/s1, Figure S1: Intercellular CellPhoneDB analysis illustrating expression of the ligand–receptor complexes among the indicated cell types in plaque MF. Figure S2: Intercellular CellPhoneDB analysis illustrating expression of the ligand–receptor complexes among the indicated cell types in tumor MF.
