# Supplementary figures and images for: Clinical Response to Anti-CD47 Immunotherapy Is Associated with Rapid Reduction of Exhausted Bystander CD4+ BTLA+ T Cells in Tumor Microenvironment of Mycosis Fungoides
Source: Cancers (Basel). 2021 Nov 28;13(23):5982. doi: 10.3390/cancers13235982 (PMC8656720; doi:10.3390/cancers13235982)

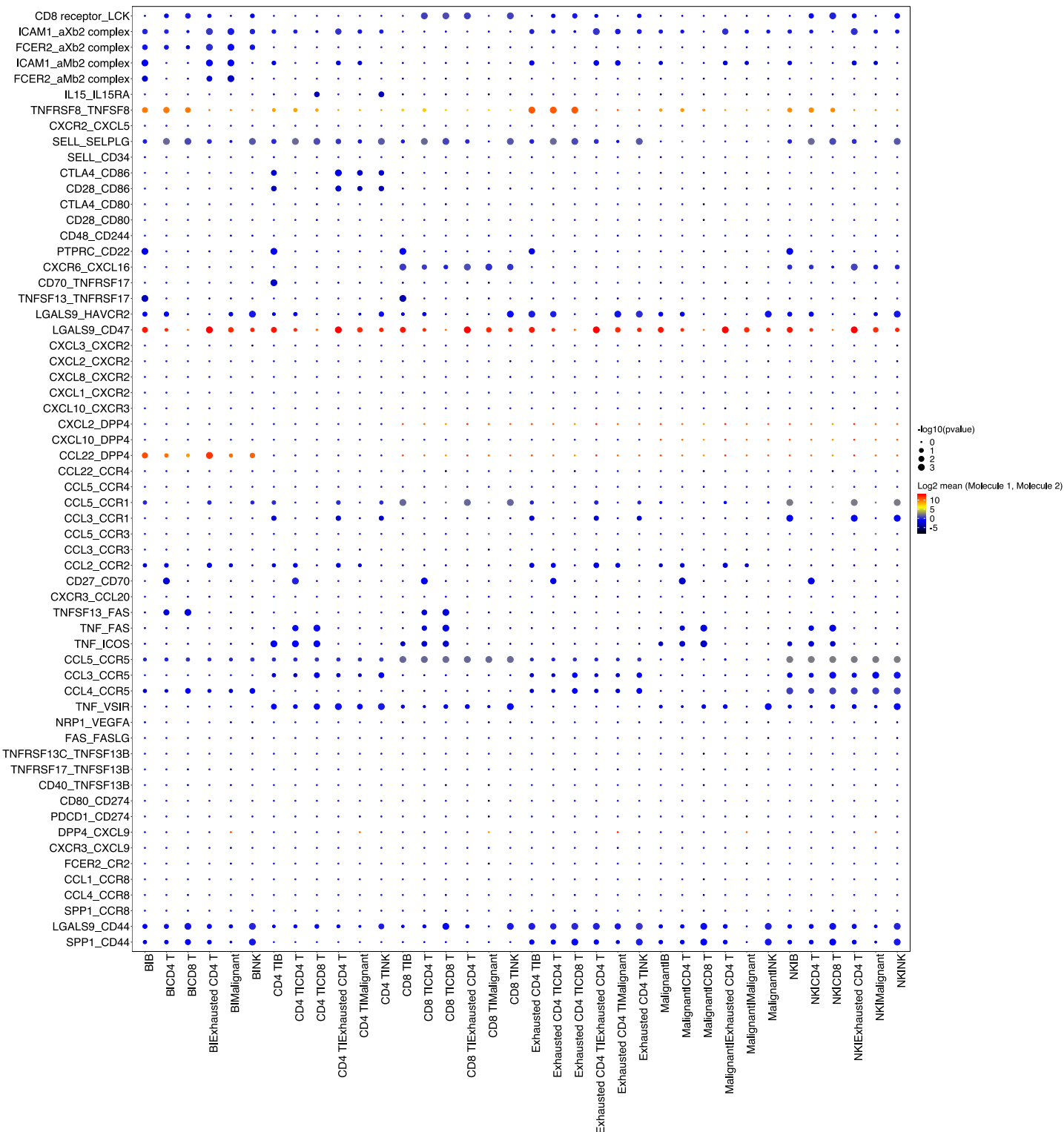

Supplemental Figure S1

Supplement: Supplementary file 1 [file cancers-13-05982-s001.zip › cancers-1464896-supplementary.pdf]
